# Progress in the treatment of diabetic macular edema with faricimab: a review

**DOI:** 10.3389/fmed.2025.1682311

**Published:** 2025-10-14

**Authors:** Mingxue Gao, Chunmeng Liu, Yining Zeng, Xiaoxiao Wu, Junguo Duan

**Affiliations:** ^1^Chengdu University of TCM, Chengdu, Sichuan, China; ^2^Eye College of Chengdu University of TCM, Chengdu, Sichuan, China; ^3^Ineye Hospital of Chengdu University of TCM, Chengdu, Sichuan, China; ^4^Eye Health with Traditional Chinese Medicine, Key Laboratory of Sichuan Province, Chengdu, Sichuan, China

**Keywords:** diabetic macular edema, faricimab, VEGF-A, angiopoietin-2, bispecific antibody, extended dosing, clinical trials

## Abstract

Diabetic macular edema (DME), a prevalent complication of diabetic retinopathy, is a leading cause of vision loss among working-age individuals worldwide. It is characterized by chronic vascular leakage, inflammation, and disruption of the blood–retinal barrier, resulting in macular fluid accumulation. Anti-vascular endothelial growth factor A (VEGF-A) therapies, such as ranibizumab and aflibercept, have significantly improved visual outcomes; however, limitations such as frequent injections, persistent edema, and suboptimal responses continue to pose challenges in clinical practice. Faricimab is the first bispecific monoclonal antibody designed to concurrently inhibit VEGF-A and angiopoietin-2 (Ang-2), two key regulators of vascular permeability and inflammation. Through its dual-targeting mechanism, faricimab enhances vascular stability, reduces leakage, and enables extended treatment intervals. Phase III clinical trials (YOSEMITE and RHINE) have demonstrated its noninferior efficacy compared to aflibercept, with a substantial proportion of patients achieving dosing intervals up to 16 weeks. Emerging real-world data further support its effectiveness and durability, particularly in individuals refractory to conventional anti-VEGF agents. This review summarizes the current evidence regarding faricimab’s molecular mechanisms, pharmacokinetic profile, clinical efficacy, real-world applications, and safety. By alleviating treatment burden and supporting individualized management, faricimab represents a promising advancement in the long-term care of DME. Future research should focus on its long-term safety, identification of response biomarkers, and integration with imaging-guided algorithms to refine personalized treatment strategies.

## 1 Introduction

Diabetic macular edema (DME) is a common and potentially vision-threatening microvascular complication of diabetic retinopathy (DR), which can occur at any stage of the disease. It is characterized by breakdown of the blood–retinal barrier and increased vascular permeability, leading to fluid accumulation in the macular region and subsequent central vision impairment (1).

DR remains one of the leading causes of acquired visual disability in the working-age population worldwide. By 2045, its global prevalence is projected to reach approximately 160 million people. The estimated incidence of DME is 5.47%, with regional variations ranging from 5.14% in high-income countries to 5.81% in low-income settings (2, 3). Because the fovea contains the highest density of photoreceptors and mediates central vision, DME frequently leads to substantial functional impairment. Timely resolution of macular edema may result in visual recovery, whereas persistent or chronic edema can cause irreversible retinal damage (4, 5).

Intravitreal injection of vascular endothelial growth factor (VEGF) inhibitors remains the current gold standard for the management of DME. Agents such as ranibizumab and aflibercept have demonstrated robust efficacy in improving best-corrected visual acuity (BCVA) in randomized clinical trials and are widely adopted in routine clinical practice (6). Ranibizumab, a recombinant humanized monoclonal antibody fragment (Fab), selectively binds to all isoforms of VEGF-A, thereby blocking downstream receptor activation. Its smaller molecular size, compared to bevacizumab, enables improved retinal penetration and faster clearance (7). Aflibercept, a recombinant fusion protein, consists of the extracellular domains of VEGF receptors 1 and 2 fused to the Fc portion of human IgG1. It functions as a soluble decoy receptor capable of binding VEGF-A, VEGF-B, and placental growth factor, thereby exerting broader antiangiogenic effects (8). These agents differ in structure, binding affinity, and pharmacokinetic properties, which may influence their clinical performance in DME management (9).

Despite their efficacy in controlled trials, anti-VEGF therapies often yield suboptimal outcomes in real-world settings. Primary or secondary nonresponse, tachyphylaxis, and recurrent edema after treatment interruption remain common challenges. Moreover, the requirement for frequent intravitreal injections imposes substantial economic, logistical, and psychological burdens on patients and healthcare systems. These factors can negatively impact adherence, ultimately compromising treatment outcomes (10). Notably, even among patients receiving six or more injections per year, approximately 65.6% continue to exhibit persistent macular edema (11).

Emerging evidence indicates that, in addition to VEGF, angiopoietin-2 (Ang-2) plays a pivotal role in the pathophysiology of DME. While VEGF promotes neovascularization and increases vascular permeability, Ang-2 contributes to vascular destabilization by antagonizing the Tie-2 receptor pathway, inducing pericyte loss, and amplifying inflammatory responses (12). Importantly, Ang-2–mediated vascular leakage can occur independently of VEGF signaling, potentially accounting for the suboptimal responses observed with anti-VEGF monotherapies alone (13). The synergistic effects of VEGF-A and Ang-2 on microvascular leakage and inflammation provide a strong rationale for dual-pathway inhibition as a more comprehensive therapeutic strategy (14).

Faricimab is the first bispecific monoclonal antibody developed to concurrently neutralize both VEGF-A and Ang-2. It is engineered using CrossMAb technology, which facilitates intravitreal delivery while preserving molecular stability and activity. Faricimab has received regulatory approval for the treatment of both DME and neovascular age-related macular degeneration (nAMD) (15). Clinical trials have demonstrated that faricimab achieves visual and anatomical outcomes comparable to those of standard anti-VEGF therapies, with the added benefit of enabling extended dosing intervals of up to 12 or 16 weeks. This may significantly reduce treatment burden and improve adherence in long-term care settings (16–18).

This review provides a comprehensive overview of current evidence on faricimab in the management of DME, including its dual-target molecular mechanism, pharmacokinetic profile, clinical efficacy, real-world performance, and safety data. We also discuss future directions for optimizing faricimab-based therapy through personalized dosing strategies and imaging-guided treatment paradigms.

## 2 Methods

We carried out a systematic search to identify studies on faricimab and DME. We used PubMed, Embase, and Web of Science. The search covered publications from January 2010 to June 2025. Search terms included *“faricimab,” “diabetic macular edema (DME),” “diabetic retinopathy (DR),” “vascular endothelial growth factor (VEGF),”* and *“angiopoietin-2 (Ang-2).”* We combined these terms with Boolean operators.

### 2.1 Eligibility criteria

We included original studies, clinical trials, observational research, and reviews. Each study had to report on the mechanism, pharmacokinetics, efficacy, safety, or real-world use of faricimab. We excluded case reports, case series with fewer than 20 patients, conference abstracts without full text, and animal studies. Relevant preclinical findings were summarized separately when needed.

### 2.2 Study selection and appraisal

Two reviewers screened all titles and abstracts. We retrieved the full text for studies that met the inclusion criteria. Disagreements were resolved through discussion. We did not perform a formal bias assessment or a meta-analysis. Instead, we considered study design, sample size, follow-up time, and outcome measures to guide interpretation.

## 3 Molecular mechanisms of Faricimab

### 3.1 Inhibition of VEGF-A

In the pathogenesis of DME, chronic hyperglycemia activates multiple biochemical pathways—oxidative stress, inflammation, and hypoxia—all of which contribute to the upregulation of VEGF-A (19). VEGF-A promotes endothelial permeability and neovascularization primarily through binding to VEGF receptor 2 (VEGFR2), which initiates downstream PI3K/Akt and MAPK signaling cascades (20, 21). Persistent VEGF-A overexpression disrupts the blood–retinal barrier, facilitating fluid accumulation in the macula.

Faricimab binds VEGF-A with high specificity and affinity, competitively inhibiting its interaction with VEGFR2 and suppressing pathological vascular permeability and neovascular proliferation. In addition, VEGFR2-mediated phosphorylation of vascular endothelial cadherin (VE-cadherin) leads to disassembly of endothelial junctions; faricimab mitigates this process and helps stabilize the vascular barrier (22).

### 3.2 Antagonism of Ang-2

Despite adequate VEGF-A inhibition, more than 30% of patients exhibit persistent edema, implicating alternative mechanisms such as the Ang–Tie-2 signaling axis (23). Angiopoietin-1 (Ang-1), produced by perivascular cells, activates the Tie-2 receptor on endothelial cells to maintain vascular quiescence, promote pericyte survival, and suppress inflammation (24, 25).

In contrast, Ang-2 is upregulated under hyperglycemic and inflammatory conditions. Secreted by endothelial cells, it competitively inhibits Ang-1–mediated Tie-2 signaling, leading to endothelial destabilization, pericyte dropout, leukocyte adhesion, and proinflammatory cytokine release (12, 26). Elevated intraocular Ang-2 levels have been observed in DR and DME, making it a critical therapeutic target (27).

### 3.3 Dual targeting to modulate inflammation and oxidative stress

VEGF-A and Ang-2 act through distinct but synergistic pathways to exacerbate microvascular dysfunction. Co-expression of both factors amplifies endothelial proliferation, vascular leakage, oxidative stress, and inflammation (28). Simultaneous inhibition of VEGF-A and Ang-2 offers a more comprehensive approach by targeting multiple aspects of the disease cascade.

Mechanistically, faricimab exerts therapeutic effects through three primary actions:

(1) Reduction of vascular leakage via inhibition of VEGF-A/VEGFR2 signaling;
(2) Suppression of inflammation by restoring Tie-2–mediated vascular stability;
(3) Stabilization of the blood–retinal barrier by preventing VE-cadherin disassembly and endothelial junction disruption.

**Figure 1:**
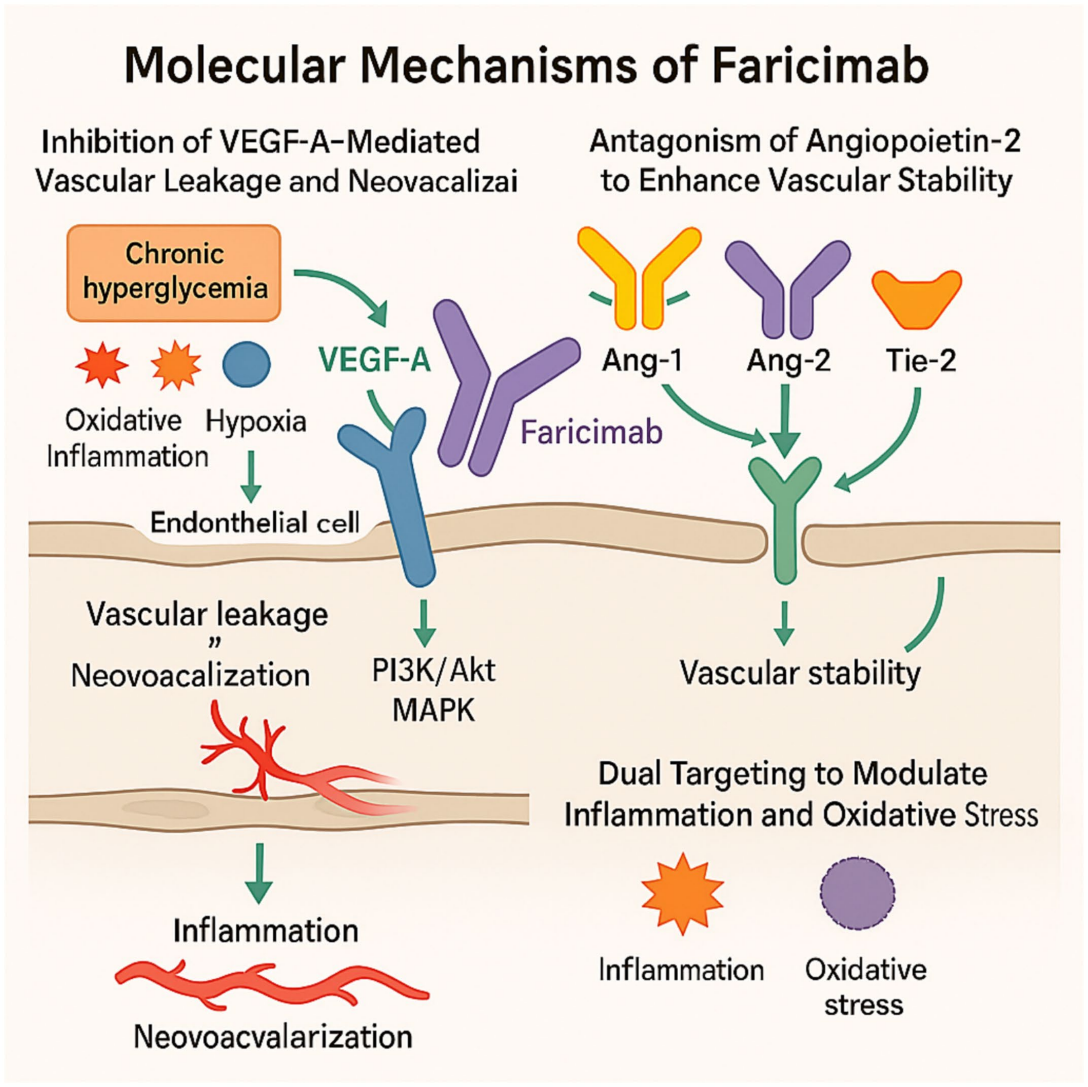
Mechanism of action of faricimab, created with BioRender.com.which targets VEGF-A and Ang-2, in DME. Hyperglycemia and hypoxia upregulate VEGF-A, increasing vascular permeability and neovascularization through VEGFR2 activation. Meanwhile, Ang-2 impairs Tie-2 signaling, promoting vascular leakage and inflammation. Faricimab, a bispecific antibody, simultaneously blocks both VEGF-A and Ang-2, thereby reducing vascular leakage, suppressing inflammation, and stabilizing the blood–retinal barrier.

The proposed mechanism of action is illustrated in Figure 1.

### 3.4 CrossMab technology and bispecific design

Faricimab is developed using CrossMab technology, which enables the incorporation of two distinct single-chain variable fragments targeting VEGF-A and Ang-2 within a single antibody structure. This platform ensures stable folding, functional integrity, and manufacturability while allowing intravitreal administration (29, 30).

By co-targeting VEGF-A and Ang-2, faricimab delivers a multifaceted therapeutic effect that addresses both vascular permeability and inflammatory dysregulation. The Ang-1/Ang-2 ratio in aqueous humor has been proposed as a potential biomarker for treatment response to faricimab, with lower ratios associated with residual edema (31).

This dual-action mechanism underpins faricimab’s therapeutic rationale in DME.

## 4 Pharmacokinetics of faricimab

### 4.1 Intraocular target suppression dynamics

Faricimab is a bispecific monoclonal antibody with an engineered Fc region designed to prolong intraocular residence time while minimizing systemic exposure (32).

Population pharmacokinetic analyses from phase II and III trials demonstrated that intravitreal faricimab provides sustained suppression of both VEGF-A and Ang-2. Aqueous humor sampling showed that Ang-2 concentrations remained below the lower limit of quantification (LLOQ) in 79% of samples at 8 weeks and 55% at 12 weeks after injection. In contrast, VEGF-A suppression was less durable, with only 7% of samples remaining below the LLOQ at 12 weeks.

These results highlight a key pharmacodynamic feature: Ang-2 inhibition persists longer than VEGF-A, suggesting differential target kinetics. Pharmacokinetic modeling indicates that faricimab maintains >50% inhibition of both targets under 4- to 8-week dosing intervals. Ang-2 suppression may extend to 12–16 weeks, while VEGF-A levels generally rebound by weeks 9–10 (33, 34).

Table 1 summarizes the target suppression durations observed in clinical studies.

**Table 1:** Duration of intraocular molecular target suppression following intravitreal faricimab, based on pharmacokinetic analyses in DME patients.

Target	Approximate suppression duration	% of samples below LLOQ at week 12
Ang-2	Up to 12–16 weeks	55%
VEGF-A	~8–10 weeks	7%

LLOQ, Lower Limit of Quantification; DME, Diabetic Macular Edema; Ang-2, Angiopoietin-2; VEGF-A, Vascular Endothelial Growth Factor A.

### 4.2 Systemic exposure and safety

Systemic exposure to faricimab is minimal following intravitreal administration. Plasma levels are approximately 1/6000th of vitreous concentrations, indicating a low likelihood of systemic adverse events.

Moreover, phase III trials demonstrated comparable efficacy across dosing intervals ranging from 8 to 16 weeks, supporting its pharmacokinetic suitability for flexible and individualized treatment strategies, such as the Personalized Treatment Interval (PTI) approach (33, 34).

### 4.3 Post-vitrectomy considerations

Following vitrectomy, the absence of the vitreous gel significantly accelerates intraocular drug clearance, shortening the half-life of anti-VEGF agents (35, 36). A case report of neovascular AMD refractory to standard therapies suggested that faricimab remained effective even in vitrectomized eyes, likely due to its dual-target design and prolonged activity. These observations highlight the potential advantages of faricimab in complex pharmacokinetic scenarios (37).

Differential suppression durations may inform personalized treatment interval titration, enabling clinicians to tailor dosing schedules based on the more sustained inhibition of Ang-2 versus the more transient suppression of VEGF-A. This approach may optimize clinical outcomes while minimizing treatment burden.

## 5 Clinical research

### 5.1 Preclinical studies

In preclinical investigations, faricimab (formerly RG7716) demonstrated superior therapeutic efficacy compared to monotherapies by concurrently targeting VEGF-A and Ang-2. In animal models of diabetic retinopathy and choroidal neovascularization, faricimab significantly reduced vascular leakage, inflammatory infiltration, neuronal apoptosis, and retinal edema, underscoring its synergistic anti-inflammatory and neuroprotective potential (38). In the JR5558 spontaneous neovascular mouse model, it suppressed macrophage recruitment and vascular permeability more effectively than single-target agents. Similarly, in a primate model, it produced greater reductions in leakage and intraocular interleukin-6 levels than ranibizumab (39, 40).

### 5.2 Phase II clinical trial

The BOULEVARD trial (NCT02699450) was a randomized, double-masked, multicenter study evaluating faricimab in patients with treatment-naïve or previously treated disease. At week 24, faricimab produced significantly greater visual gains than ranibizumab. Secondary outcomes, including reductions in central subfield thickness (CST) and *diabetic retinopathy severity scale* (DRSS) scores, also favored faricimab. The incidence of intraocular inflammation was low and comparable between groups (41).

### 5.3 Phase III YOSEMITE and RHINE trials

The YOSEMITE (NCT03622580) and RHINE (NCT03622593) trials were identically designed, large-scale, randomized studies enrolling over 1,600 treatment-naïve patients. Participants received faricimab every 8 weeks, faricimab with a PTI, or aflibercept every 8 weeks. Faricimab demonstrated noninferior efficacy with the added benefit of extended dosing intervals: 63% of patients in the PTI group with early leakage resolution maintained 16-week intervals at week 52, while 45% of those with persistent leakage also achieved interval extension (42, 43). Subgroup analyses confirmed consistent outcomes across ethnic groups, including Asian populations (44).

Subgroup analyses confirmed consistent efficacy across ethnic groups, including Asian populations (45, 46). A complementary phase III study supported the noninferiority of faricimab using a treat-and-extend (T&E) approach, achieving durable efficacy over 2 years (47).

These phase III studies provide strong evidence for faricimab. They also have important limitations. Most participants came from Western countries, and patients with serious comorbidities were excluded. This limits the generalizability of the results. The follow-up lasted only 2 years, which is short for evaluating long-term safety. The trials defined disease activity mainly by leakage status and CST. Other studies used BCVA or microaneurysm changes. These differences can influence treatment interval decisions and make cross-trial comparisons difficult.

### 5.4 Real-world studies

Real-world evidence from larger prospective studies supports the clinical utility of faricimab in the management of DME, particularly in terms of extended treatment durability and microvascular stabilization.

The ongoing phase IV ELEVATUM trial (NCT05224102), specifically designed for treatment-naïve patients, has shown early and sustained improvements in both BCVA and CST under a structured loading and extension protocol (48).

A retrospective multicenter analysis involving 116 patients with either nAMD or DME found that faricimab improved anatomical outcomes and enabled extended treatment intervals in patients who were previously unresponsive to anti-VEGF therapy (49).

These results are further corroborated by additional small-scale studies reporting dosing interval extension and visual/anatomical benefits in patients with suboptimal responses to ranibizumab or aflibercept (50–52).

Moreover, a prospective observational study reported significant microvascular improvements, including regression of microaneurysms and enhanced retinal perfusion, suggesting a potential role for faricimab in reversing diabetic microangiopathy (53).

Despite these findings, real-world studies remain limited by small sample sizes, short follow-up, and heterogeneous design. Larger, longer-term studies are warranted to confirm the generalizability of faricimab’s benefits (Table 2).

**Table 2:** Overview of key studies evaluating faricimab in diabetic macular edema.

Study Dimension	BOULEVARD (Phase II)	YOSEMITE and RHINE (Phase III)	ELEVATUM (Phase IV, ongoing)
Design	Randomized, double-masked	Randomized, noninferiority	Prospective, observational
Population	Naïve or previously treated	Treatment-naïve	Treatment-naïve
Control	Ranibizumab 0.3 mg	Aflibercept 2.0 mg	None
Regimen	Faricimab Q4W vs. control	Faricimab Q8W or PTI vs. control	Structured loading + extension
Follow-up	24 weeks	96–100 weeks	Ongoing
Sample Size	229	>1,600 (combined)	N/A
Primary Endpoints	BCVA, CST, DRSS	BCVA, CST, % at Q12–16 W	BCVA, CST
Key Findings	Superior BCVA and CST outcomes	Noninferior efficacy; extended intervals in >60%	Early and sustained gains
Safety Profile	Inflammation <1%; well tolerated	No new safety signals	No major adverse events
Reference	(41)	(42–44)	(48)

BCVA, best-corrected visual acuity; CST, central subfield thickness; DRSS, diabetic retinopathy severity scale; MA, microaneurysms; IOP, intraocular pressure; QXW, dosing every X weeks.

### 5.5 Safety of faricimab

Faricimab, a bispecific monoclonal antibody targeting VEGF-A and Ang-2, has been extensively evaluated for safety in both clinical trials and real-world settings. Across phase II and III studies, the most commonly reported ocular adverse events following intravitreal injection included vitreous floaters, eye pain, and conjunctival hemorrhage. These events were generally mild to moderate in severity and occurred at low frequencies. Serious ocular complications were uncommon (42, 47, 54, 55). A large-scale retrospective analysis involving 1,860 patients (2,318 eyes; 10,297 injections) reported a 0.19% per-injection rate of intraocular inflammation, the majority of which were mild and self-limited (56). Another study documented a 0.6% rate of more severe intraocular inflammation, including moderate vitritis, with a few cases progressing to irreversible vision loss (57).

Of particular concern are isolated case reports linking severe intraocular inflammation to herpes simplex virus reactivation. Clinical presentations included dendritic keratitis, elevated intraocular pressure (IOP), and favorable responses to antiviral therapy (58).

A more severe manifestation involves immune-mediated delayed hypersensitivity reactions upon re-exposure, occasionally resulting in occlusive retinal vasculitis and permanent vision loss (59). In a 22-month Swiss observational study, 7 patients (12 eyes) developed noninfectious intraocular inflammation after faricimab injections; among them, 2 eyes experienced retinal vasculitis with subsequent irreversible visual decline (60).

Regarding systemic safety, a meta-analysis found no significant increase in the risk of acute kidney injury with faricimab use, with renal adverse events reported in 0.8% of patients—comparable to rates seen with other anti-VEGF agents (61). Similarly, no elevation in cardiovascular or thromboembolic risk has been observed, consistent with faricimab’s limited systemic exposure following intravitreal injection (42, 47). However, isolated case reports have described intraocular inflammation and retinal vascular occlusion, sometimes after a second injection, leading to visual deterioration (62).

Intraocular pressure elevation has also been reported, typically mild and transient. However, repeated anti-VEGF injections may impair aqueous humor outflow and increase the risk of secondary glaucoma (63). Accordingly, regular IOP monitoring is recommended during faricimab therapy, especially in patients with glaucoma or a history of ocular hypertension. Cases of hypertensive uveitis following faricimab administration further underscore the need for vigilance regarding IOP and inflammatory signs (64).

In special populations, faricimab’s high molecular weight (~149 kDa) suggests limited transfer into breast milk. Nonetheless, due to the immaturity of the neonatal gastrointestinal barrier, its use is not currently recommended during lactation (65).

Data from the U. S. FDA Adverse Event Reporting System (FAERS) have identified post-marketing reports of adverse events such as blindness, cerebral infarction, retinal hemorrhage, dry eye, and unilateral blindness, alongside labeled events including intraocular inflammation, elevated IOP, retinal pigment epithelium (RPE) tears, vitreous floaters, and retinal vascular occlusion (66). These findings highlight the importance of ongoing pharmacovigilance.

A recent case series further described suspected herpes simplex virus (HSV) reactivation following faricimab, including dendritic ulcers, granulomatous keratic precipitates, and ocular hypertension. Notably, many affected eyes did not return to baseline visual acuity (58). Although the mechanism remains unclear, it may involve immune dysregulation secondary to dual VEGF-A and Ang-2 inhibition.

Regarding drug handling, an *in vitro* study demonstrated that faricimab remains chemically and physically stable when stored in prefilled polypropylene syringes (with or without silicone oil) at 2–8 °C for up to 28 days. The drug retained binding activity and sterility, supporting the feasibility of short-term off-label preloading (67).

Overall, faricimab’s ocular safety profile is broadly comparable to other anti-VEGF agents, such as aflibercept. However, its dual-target design may theoretically affect broader biological pathways, and long-term safety data remain limited. Continued surveillance and large-scale, prospective studies are essential to fully characterize the long-term risk profile in diverse patient populations. To facilitate rapid clinical reference, a summary of adverse events and safety considerations is provided in Table 3.

**Table 3:** Summary of faricimab-associated adverse events.

Adverse event	Frequency	Severity	Comments
Intraocular inflammation	0.19–0.6%	Mostly mild	Few severe cases, rare vision loss
HSV reactivation	Rare	Severe if untreated	Responds to antiviral therapy
IOP elevation	Occasional	Mild to moderate	Monitor routinely

HSV, herpes simplex virus; IOP, intraocular pressure.

## 6 Limitations

This review has several limitations. First, it is not a meta-analysis and does not include a quantitative synthesis of outcomes across studies. While the narrative format provides flexibility in highlighting mechanistic and clinical nuances, it may be prone to selection bias. Second, many of the cited studies, especially real-world data, are heterogeneous in design, with variations in sample size, treatment protocols, and follow-up duration. Third, several promising results—including retinal microvascular improvement and dosing durability—are derived from short-term studies or early-phase trials, which require further validation through long-term data.

In addition, the generalizability of certain findings remains uncertain in specific populations. For example, some efficacy and safety outcomes have not been robustly evaluated in Asian cohorts or underrepresented ethnic groups. Finally, the absence of Artificial Intelligence-assisted imaging analysis in current treatment protocols limits the integration of quantitative optical coherence tomography angiography (OCTA) parameters into routine clinical decision-making.

## 7 Conclusion and future perspectives

Faricimab represents a novel and promising advancement in the management of DME through its dual inhibition of VEGF-A and Ang-2. This bispecific approach not only improves anatomical and functional outcomes but also enables extended dosing intervals—up to 16 weeks in many cases—offering an alternative to frequent intravitreal injections. Clinical trials and real-world studies have consistently confirmed its efficacy and safety, particularly among patients who respond inadequately to conventional anti-VEGF monotherapies.

The PTI model allows for tailored dosing based on disease activity, reducing injection frequency and clinic visits. This flexibility may alleviate treatment fatigue, improve adherence, and optimize healthcare resource utilization. In doing so, faricimab contributes to lowering the socioeconomic burden associated with chronic retinal diseases—particularly in aging populations and resource-constrained healthcare systems—by minimizing the need for frequent appointments, travel, and caregiver involvement.

However, while short-term outcomes are encouraging, long-term data on structural preservation—such as nerve fiber layer integrity, capillary perfusion, and sustained visual function—are still lacking. Further studies with extended follow-up and diverse populations are essential to validate the durability and generalizability of faricimab’s benefits. Notably, many pivotal trials have predominantly enrolled Western patients, leaving the treatment response in Asian and other underrepresented populations less well characterized.

Looking forward, the integration of artificial intelligence with retinal imaging tools such as optical coherence tomography (OCT) and OCTA may enhance early detection of microvascular changes, stratify patient risk, and guide interval titration. For example, artificial intelligence (AI)-based quantification of ischemia, microaneurysm dynamics, and perfusion density could enable proactive treatment adjustments before irreversible damage occurs. Additionally, innovations such as sustained-release intravitreal implants may further extend dosing intervals while maintaining efficacy, potentially transforming maintenance therapy paradigms.

Taken together, current evidence indicates that faricimab combines a dual-target mechanism with dosing flexibility, and has been associated with improved durability and favorable anatomical outcomes in clinical studies. Its potential for long-term disease control requires further validation in real-world settings.
